# Iodine nutritional status and risk factors for goitre among schoolchildren in South Tajikistan

**DOI:** 10.1186/1472-6823-13-50

**Published:** 2013-11-02

**Authors:** Barbara Matthys, Mohbegim Davlatmamadova, Gulzira Karimova, Vreni Jean-Richard, Michael B Zimmermann, Kaspar Wyss

**Affiliations:** 1 Swiss Tropical and Public Health Institute, Swiss Centre for International Health, P.O. Box, CH-4002, Basel, Switzerland; 2 University of Basel, P.O. Box, CH-4003, Basel, Switzerland; 3 Republican Clinical Endocrinology Centre, 734013 House No.7, 2nd drive, str. Zehni T, Dushanbe, Tajikistan; 4 Project Sino, House No 32, Akademika Adhamova, 734024 Dushanbe, Tajikistan; 5 Swiss Tropical and Public Health Institute, Department of Epidemiology and Public Health, P.O. Box, CH-4002, Basel, Switzerland; 6 Swiss Federal Institute of Technology Zürich, Laboratory for Human Nutrition, ICCIDD Global Network, Schmelzbergstr. 7, 8092 Zürich, Switzerland

**Keywords:** Iodine status, Goitre, Risk factors for goitre, Urinary iodine concentration, Thyroglobulin concentration, Salt iodization, Schoolchildren, Tajikistan

## Abstract

**Background:**

Iodine deficiency affects nearly 1.9 billion people worldwide, but it can be prevented by salt iodization. This cross-sectional survey assessed current iodine status, iodized salt coverage and risk factors for goitre among schoolchildren in South Tajikistan.

**Methods:**

Ten primary schools in four districts in South Tajikistan were randomly selected. In schoolchildren aged 7 to 11 years, a spot urine sample was collected for measurement of urinary iodine, dried blood spots were collected for measurement of thyroglobulin, and goitre was assessed by palpation. Iodine content of salt samples and local selling points was determined by coloration using rapid test kits and titration method.

**Results:**

Of 623 schoolchildren enrolled, complete data was obtained from 589. The overall median urinary iodine concentration (UIC) was 51.2 μg/L indicating mild-to-moderate iodine deficiency. Among all children, 46.6% (95% Confidence Interval (CI) = 42.4%-50.6%) of children were found to be goitrous (grade 1 goitre: 30.6%, 95% CI = 26.9%-34.5%; grade 2 goitre: 16.0%, 95% CI = 13.1%-19.2%). The risk factor for goitre remaining significant in the multivariable logistic regression model was 'buying salt once a month’ (OR = 2.89, 95% CI = 1.01-8.22) and 'buying salt once every six months’ (OR = 2.26, 95% CI = 1.01-5.04) compared to 'buying salt every one or two weeks’. The overall median thyroglobulin concentration was elevated at 13.9 μg/L. Of the salt samples from households and selling points, one third were adequately iodised, one third insufficiently and one third were not iodised.

**Conclusion:**

Iodine deficiency remains a serious health issue among children in southern Tajikistan. There is a persisting high prevalence of goitre, elevated thyroglobulin and low UIC despite interventions implemented by Tajikistan and international partners. Quality control of salt iodine content needs to be improved. Continued efforts to raise awareness of the health effects of iodine deficiency are needed to increase consumer demand for iodised salt.

## Background

Approximately 1.88 billion people worldwide have inadequate iodine intake, including one third of all school-aged children [1,2]. Iodine deficiency has serious adverse effects on growth and development of humans, such as mental impairment [3]. However, some global trends of improvement since 2003 have recently been observed [2]. An effective strategy to prevent iodine deficiency disorders (IDD) is salt iodisation.

Iodine deficiency in children is assessed and monitored by three recommended methods that are complementary: 1) urinary iodine concentration (UIC) is an indicator of recent iodine intake over the previous 1–2 days, as up to 90% of iodine is absorbed and excreted in the urine; 2) thyroglobulin, a thyroid-specific protein that is secreted into the bloodstream during iodine deficiency and reflects iodine status over weeks to months; and 3) changes in the goitre rate over months to years are assessed by the size of the thyroid gland [4,5].

The independence of the Central Asian region from the Soviet Union in 1991 and the subsequent civil war in Tajikistan led to discontinuation of salt iodation programs and collapse of health care systems, resulting in an increase of iodine deficiency disorders [6,7].

The government of the Republic of Tajikistan designed legislation on salt iodation and launched the National Programme for Elimination of IDD in 1997. The law “On salt iodization”, regulating the production, distribution and consumption of iodized salt, was adopted in 2002 [8]. The National Programme was implemented by specified institutions such as the Republican Clinical Endocrinology Centre, the State Sanitary Epidemiological Surveillance Service (SSESS) and the Healthy Lifestyle Centre, and supported by international partners, as for example UNICEF and the Aga Khan Foundation. At district level, school canteens are provided with iodized salt, schoolchildren are screened annually for goitre, and iodine content in cooking salt samples of selling points and households are monitored by family doctors using iodine rapid tests.

Community groups identified iodine deficiency as a priority health issue. Therefore, the iodine status of schoolchildren from selected schools in four pilot districts in southern Tajikistan was assessed by a cross-sectional study using UIC, serum thyroglobulin concentration, thyroid size and iodisation level of salt. In addition, potential risk- and protective factors for goitre were identified. The study findings were compared with published literature on IDD available from Central Asian countries.

## Methods

### Study area

The study area and context have been previously detailed, because this study jointly assessed the occurrence of helminths and intestinal protozoa in school settings [9]. In brief, the Central Asian Republic of Tajikistan counts approximately 6.9 million inhabitants [10] with three quarters living in rural areas [11]. Remittances transmitted by migrants working in Russia represent a vital income source for households according to estimations by the National Bank with 49% of the gross domestic product (GDP) whereas agriculture contributed 24% to the GDP in 2008 [12].

Climate is continental to Mediterranean with pronounced rainfall in winter and spring, hot and dry climate in summer and cold winters [13]. A major part of the territory is mountainous with predominant plains in the South. Loess deposits at foothills and plains in Tajikistan are fertile but highly erodable. Water erosion e.g. through extensive irrigation accelerated the substantial soil degradation processes and loss of organic material [14,15]. Iodine content of soils are determined primarily by proximity to the sea coast and organic content controlling iodine fixation in soils. Marine iodine from atmospheric deposition unlikely reaches inland regions [16]. Soils from landlocked and mountainous regions, flood plain areas and accumulative landscapes are known for being environmentally iodine deficient, as reported in studies from Turkey [17], China [18] and the central Russian plain [19]. A study investigating iodine content of soils in eastern Afghanistan describe the soils as sandy with low in organic material and as iodine-poor [20]. These regions were thus associated with prevalence of goitre [20,21].

### Study population

**Figure 1:**
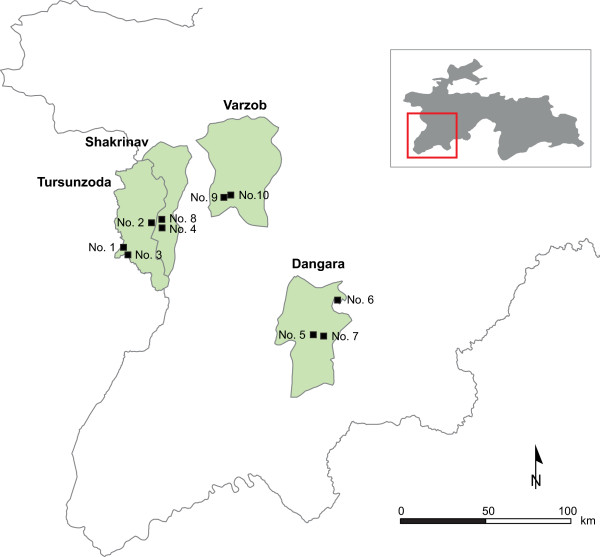
Study area of selected schools in South Tajikistan for a cross-sectional iodine status survey in 2009.

The cross-sectional study was conducted in four pilot districts of the Swiss Health Reform and Family Medicine Support Project (Project Sino in short) in the regions of Khatlon and the Republican Subordination (RRS) in South Tajikistan in 2009 (Figure 1). Based on data from the Republican Agency of Statistics of Tajikistan, the total number of population of the four pilot districts was 517’680, representing 7.0% of the total estimated population of Tajikistan in 2009 [22]. Schoolchildren aged seven to eleven years attending grades two and three were sampled because of their high vulnerability to ID, representativeness, and accessibility within the community [5]. Tajikistan is estimated to have a high school enrolment of 97% [23].

### Sample design and selection of schools

Because the sampling was designed to assess both iodine status, and helminths and intestinal protozoa, the sampling approach adhered to a rapid appraisal methodology proposed by the WHO to identify high risk groups for school-based deworming: 5–10 schools are selected within an ecologically homogenous area and a minimum of 50 schoolchildren of an age range bearing highest risk of infection per school are examined [24,25]. Lists of all primary schools with the number of children attending grades two and three were prepared by the heads of educational departments in the four districts. Schools with less than 30 children attending these two grades and ten schools from one district due to previous deworming activities were excluded. From a total of 300 listed schools, 143 having less than 50 children attending grades two and three had to be excluded. Because of recent deworming activities 10 additional schools from one district were excluded. A simple random sampling procedure was employed to select 10 out of 147 schools. Eight schools were located in lowland areas and two in mountainous valleys and were thus considered being located in the ecological lowland area. Depending on the sizes of classes, 60–70 children were chosen in each school to obtain a minimum sample size of 500 fully complying children.

### Field procedures

A questionnaire tool consisting of a household module on salt consumption patterns for caretakers and an individual module for schoolchildren was developed. The questionnaire tool was pilot tested in a school and in households in a community near Dushanbe so to train the field teams in data collection procedures and to adapt the tool to the cultural context. Directors and teachers of grades two and three from the selected schools were visited prior to field data collection. Purpose and operational procedures of the study were explained.

The household module including a written consent form for caretakers and plastic containers for a salt samples were left with the teachers to be distributed to all eligible schoolchildren. The caretakers of the schoolchildren were asked to fill the household module and to provide a handful (~20 g) of cooking salt. Salt samples from all selling points in the village, (i.e. market, grocery store, private selling points and school canteen) were collected by a voluntary teacher.

In the early morning of the survey, household questionnaires, signed informed consent sheets and salt samples were collected. The schoolchildren presented non-fasting. A unique identification number was assigned to each schoolchild participating in the study. A spot urine sample was obtained from each child into a small plastic container. Finger prick blood samples were collected on filter paper cards for the thyroglobulin assay by an experienced parasitologist from the Republican Tropical Disease Centre following standard operating procedures. An endocrinologist from the Republican Clinical Endocrinology Center determined the presence of goitre by palpation. Questions on hygiene behaviour, drinking water and sanitation based on a standard tool of the 'Joint Monitoring Programme for Water Supply and Sanitation’ developed by the WHO and UNICEF were asked to the schoolchildren [26]. Each child was weighed to the nearest kg and measured to the nearest cm. Two ml of urine was filled in a tightly-capped Eppendorf tube and transferred for analyses at the Laboratory for Human Nutrition, Swiss Federal Institute of Technology Zurich in Switzerland. The samples were stored frozen at minus 20°C until laboratory analysis.

### Laboratory procedures

#### *Quantitative examination of salt samples on iodine content*

Collected salt samples were examined semi-quantitatively for iodine content using rapid test kits provided by UNICEF Tajikistan. The teachers and directors were informed on the overall prevalence of goitre among the examined schoolchildren and the iodine level of the salt samples. One quarter of the salt samples per location were randomly selected and transferred to the laboratory of the SSESS. The samples were examined for iodine content using the quantitative titration method, which is the reference method to analyse iodine content in salt samples [5]. A modification of the Sandell-Kolthoff method was employed to measure the iodine content in the urine samples [27]. Thyroglobulin was measured in the dried blood spots using a serum dissociation enhanced lanthanide fluorescent immunoassay (DELFIA) thyroglobulin kit (hTg) which was adapted to whole dried blood spots [28], as previously described [29].

### Ethics considerations

The study was approved by the Ministry of Health (MoH) of Tajikistan (Reference letter no. 16/75-92). The study protocol was presented to collaborating partner institutes to discuss the survey methodology and procedures. The Primary Health Care Network Managers of each health district and local authorities in the communities were met to explain the study and their oral consent was obtained. A written informed consent form was signed by the caretakers of participating children before study enrolment. Children and caretakers could withdraw from the study without further obligation at any time and participation was voluntary. A prescription for treatment was provided to children with diagnosed goitre. Each child was compensated with soap and a small pack of iodine-fortified salt. At the end of the study, a debriefing meeting for local authorities was held to discuss preliminary findings.

### Data management and statistical analysis

EpiData version 3.1 (EpiData Association; Odense, Denmark) was used for single data entry. Systematic internal consistency checks were performed. Data analysis was performed in Stata version 10 (Stata Corporation; College Station, TX, USA). A household-based asset approach adapted from previous studies in Tajikistan was employed for determining the children’s socioeconomic status [30,31]. Principal component analysis methodology was used to estimate household asset weights and construct a wealth index [32]. Each child was assigned to three wealth classes (bottom, 40%; middle, 40%; top, 20%). The procedures were detailed elsewhere [9].

The iodine concentration of salt samples was categorised into 0 (no iodine), 1 (insufficiently iodised; 1–14 parts per million; ppm iodine), and 2 (adequately iodised; ≥15 ppm iodine). A median concentration of urinary iodine of 200–299 μg/L was defined as 'above requirements’, 100–199 μg/L as 'adequate’, and <100 as insufficient following standard criteria for assessing iodine nutrition based on median UI concentrations of school-aged children [5]. The category 'insufficient’ was subdivided into 'mild iodine deficiency’ (50–99 μg/L), 'moderate iodine deficiency’ (20–49 μg/L) and 'severe iodine deficiency’ (<20 μg/L). For thyroglobulin, a reference interval of 4–40 μg/L and a population median of 13 μg/L were used to classify iodine status in school-aged children [29,33].

The thyroid size was classified according to criteria recommended by the Joint WHO/UNICEF/ICCIDD Consultation, differentiating between grade 0 (non-palpable goitre), 1 (palpable but not visible goitre) and 2 (palpable and visible goitre) [5]. The classification of drinking water sources by the WHO/UNICEF JPM was employed to build groups of 'improved’ (i.e. piped water into dwelling/yard, public tap or standpipe, bottle water and rainwater) and 'unimproved’ sources (i.e. unprotected spring, cart with tank, tanker truck and surface water) [26].

Categorical data were summarised by frequencies and percentages and continuous data by mean standard deviation (SD) and quartiles. Pearson’s χ^2^-test or Fisher’s exact test were employed as appropriate to compare proportions. Nonparametric Spearman’s rank correlation coefficient was used to test correlation between urinary iodine concentration and thyroglobulin concentration, both covariates not being normally distributed. Cochran-Mantel Haenszel test was employed to assess statistical significance of associations, and non-parametric Kruskall-Wallis test for differences between medians. Bi- and multivariable logistic regression models were fitted to analyse risk factors for goitre defined by grade 1 and 2. Explanatory variables associated with goitre showing a *P*-value <0.15 in the bivariate model, i.e. 'surface water’ and 'public tap/standpipe’ (drinking water sources); 'salt bought from shop nearby town’; 'salt bought from wholesale shop’; 'plastic bag without label’ (type of salt package); 'less than 1 kg’ (quantity of salt bought last time), and 'adequately iodized’ (iodization level of salt), were included in the multivariable model. Covariates ≥ 0.15 were removed one by one following a stepwise backward elimination approach. A school-level random effect was introduced considering variations of conditions between schools. A *P*-value of ≤0.05 was considered indicative of a statistically significant difference or association and 95% confidence intervals (CIs) were calculated for the tests.

## Results

623 schoolchildren from grades two and three were registered and 602 students participated in the cross-sectional survey resulting in a compliance of 96.6%. Non-compliance was explained by absence due to travel (n = 9), absence of written informed consent (n = 3), feeling unwell (n = 2), or no specific reason (n = 7). The age range was from seven to eleven years with a mean of 9.1 years (SD = 0.73). The number of boys was slightly higher than the number of girls (311 *versus* 291, *P* = 0.416), but without differences across schools (Pearson’s χ^2^ (9) = 10.47, *P* = 0.314). Complete data for goitre, thyroglobulin, urinary iodine, and qualitatively tested salt samples were obtained from 589 schoolchildren.

### Profiles of the households

The socioeconomic and -demographic profile was described elsewhere [9]. A majority of the households included six to ten persons. Small households of three to five persons accounted for 19% and large households of more than ten persons for 8%. Farmers or workers represented two third of the household heads. Regarding educational attainment, secondary school degree was completed by every second household head and university degree by every third. Almost all households kept livestock, such as cows, donkeys, goats, sheep and chicken.

### Prevalence of goitre and risk factors

Prevalence of goitre is summarised in Table 1. Overall, goitre was diagnosed in almost every second schoolchild (46.6%; 95% CI = 42.4%-50.6%) with pronounced geographic disparities across locations of schools ranging from 19.3% (95% CI = 10.4%-31.4%) to 65.1% (95% CI = 52.0%-76.7%). Every third child (30.6%; 95% CI = 26.9%-34.5%) was affected by goitre grade 1 and every sixth child (16.0%; 95% CI = 13.1%-19.2%) by goitre grade 2. Highest occurrences of goitre grade 2 were observed in two schools (30.5%, 95% CI = 19.2%-43.9% and 27.0%, 95% CI = 16.6%-39.7%; respectively).

**Table 1:** Prevalence of goitre stratified by sex and overall prevalence in South Tajikistan, 2009

**School nr**		**Boys**			**Girls**					**Total**		
	**Nr**	**Grade 0**	**Grade 1**	**Grade 2**	**Grade 0**	**Grade 1**	**Grade 2**	**χ**^**2**^^**a**^	***P*-value**	**Grade 0**	**Grade 1**	**Grade 2**
**1**	61	22 (62.9)	10 (28.6)	3 (8.6)	14 (53.9)	8 (30.8)	4 (15.4)	NA^b^	0.704	36 (59.0)	18 (29.5)	7 (11.5)
**2**	59	17 (48.6)	13 (37.1)	5 (14.3)	12 (50.0)	9 (37.5)	3 (12.5)	NA^b^	0.999	29 (49.2)	22 (37.3)	8 (13.6)
**3**	59	13 (40.6)	13 (40.6)	6 (18.8)	9 (33.3)	6 (22.2)	12 (44.4)	NA^b^	0.091	22 (37.3)	19 (32.2)	18 (30.5)
**4**	63	10 (37.0)	11 (40.7)	6 (22.2)	12 (33.3)	13 (36.1)	11 (30.6)	NA^b^	0.810	22 (34.9)	24 (38.1)	17 (27.0)
**5**	49	5 (26.3)	13 (68.4)	1 (5.3)	18 (60.0)	7 (23.3)	5 (16.7)	NA^b^	0.008	23 (46.9)	20 (40.8)	6 (12.2)
**6**	54	18 (60.0)	8 (26.7)	4 (13.3)	16 (66.7)	6 (25.0)	2 (8.3)	NA^b^	0.921	34 (63.0)	14 (25.9)	6 (11.1)
**7**	66	16 (50.0)	9 (28.1)	7 (21.9)	19 (55.9)	9 (26.5)	6 (17.7)	NA^b^	0.849	35 (53.0)	18 (27.3)	13 (19.7)
**8**	66	13 (40.6)	14 (43.8)	5 (15.6)	14 (41.2)	12 (35.3)	8 (23.5)	NA^b^	0.692	27 (40.9)	26 (39.4)	13 (19.7)
**9**	62	33 (86.8)	4 (10.5)	1 (2.6)	17 (70.8)	6 (25.0)	1 (4.2)	NA^b^	0.331	50 (80.7)	10 (16.1)	2 (3.2)
**10**	50	21 (87.5)	1 (4.2)	2 (8.3)	16 (61.5)	8 (30.8)	2 (7.7)	NA^b^	0.042	37 (74.0)	9 (18.0)	4 (8.0)
**Total**	589	168 (55.3)	96 (31.6)	40 (13.1)	147 (51.6)	84 (29.5)	54 (19.0)	3.68	0.159	315 (53.5)	180 (30.6)	94 (16.0)
**95% CI**		(49.5, 60.9)	(26.4, 37.1)	(9.6, 17.5)	(45.6, 57.5)	(24.2, 35.1)	(14.6, 24.0)			(49.4, 57.6)	(26.9, 34.5)	(13.1, 19.2)

^a^Comparison of frequencies of 2 categories 'goitre’ and 'no goitre’ using Pearson’s χ^2^ test; ^b^ = Fisher’s exact test.

Stratified by sex, goitre was observed in 44.7% (95% CI = 39.1%-50.5%) of boys and 48.4% (95% CI = 42.5%-54.4%) of girls. Goitre grade 1 was observed in 31.6% (95% CI = 26.4%-37.1%) of boys and 29.5% (95% CI = 24.2%-37.1%) of girls and grade 2 was slightly more frequent in girls (19.0%; 95% CI = 14.6%-24.0%) compared to boys (13.1%; 95% CI = 9.6%-17.5%). Expectedly, older schoolchildren were more often suffering from goitre grade 2 than younger ones (7–8 years-old: 6.5%, 95% CI = 2.8%-12.4%; 9-years-old: 17.4%, 95% = 13.1%-22.3%; 10–11 years-old: 20.1%, 95% CI = 14.6%-26.6%; Fisher’s exact test, *P* = 0.010).

An association with marginal significance between goitre grades and salt iodization across schools was observed: goitre grade 2 was observed less frequently in children with an adequately iodised salt sample from their household (15.5%; 95% CI = 10.7%-21.3%) when compared to children with a non-iodised salt sample from their household (20.6%, 95% CI = 15.2%-27.0%; Pearson’s χ^2^ (4) = 9.585, *P* = 0.048).

Findings from bivariate logistic regression models (Table 2) pointed out that potential risk factors for goitre were 'buying salt from a wholesale shop’ (OR = 1.98, 95% CI = 1.14 - 3.43) compared to 'buying salt from a local shop in the village or a nearby town’, 'buying salt every 2–3 months’ (OR = 1.76, 95% CI = 1.04-2.98) compared to 'buying salt every 1–2 weeks’, 'buying quantities of salt more than 5 kg’ (OR = 2.24, 95% CI = 1.06-4.72) compared to 'buying quantities of salt less than 1 kg’, and a thyroglobulin concentration of >40 μg/L (OR = 1.78, 95% CI = 1.14-2.81). A protective factor was the iodisation level of salt (inadequately iodised < 15 ppm: OR = 0.56, 95% CI = 0.38 - 0.84). Specific drinking water sources i.e. 'public tap/standpipe’ turned out as a protective factor (OR = 0.39, 95% CI = 0.27, 0.55) compared to 'unprotected wells and surface water’. Socioeconomic status, i.e. children from middle class households (OR = 0.71, 95% CI = 0.49 - 1.02) and buying frequently salt once a week or once every two weeks (OR = 0.67, 95% CI = 0.44 - 1.03) were almost significant protective factors.

**Table 2:** Risk factors for goitre in schoolchildren resulting from logistic regression models in South Tajikistan, 2009

	**Goitre**					
	**Bivariate model**^**a**^			**Multivariable model**^**b**^		
**Explanatory variables**	**OR**	**(95% CI)**	**P-value**^**c**^	**OR**	**95% CI**	**P-value**^**c**^
Demography						
Sex						
Male	1.00					
Female	1.16	(0.84, 1.60)	0.370			
Age (years)						
7-8	1.00					
9	1.11	(0.73, 1.71)				
10-11	1.32	(0.83, 2.09)	0.462			
Location of school						
1	1.00					
2	1.49	(0.72, 3.07)				
3	2.42	(1.16, 5.05)				
4	2.68	(1.30, 5.55)				
5	1.63	(0.76, 3.48)				
6	0.85	(0.40, 1.80)				
7	1.28	(0.63, 2.58)				
8	2.08	(1.02, 4.22)				
9	0.35	(0.15, 0.78)				
10	0.51	(0.22, 1.14)	<0.001			
Socioeconomic status						
Bottom 40%	1.00					
Middle 40%	0.71	(0.49, 1.02)				
Top 20%	0.77	(0.49, 1.19)	0.159			
Meat consumption per week						
< 1 times	1.00					
1-2 times	0.99	(0.66, 1.48)				
3-4 times	1.26	(0.75, 2.11)				
> 4 times	0.81	(0.45, 1.47)	0.596			
Source of drinking water						
Unprotected well, surface water	1.00					
Piped water in dwelling	0.61	(0.33, 1.14)				
Water tap in yard (shared with neighbours)	0.53	(0.25, 1.14)				
Public tap/standpipe	0.39	(0.27, 0.55)	<0.001			
Infection with single or multiple species pathogenic parasite^d^	1.17	(0.83, 1.63)	0.368			
Salt bought from						
Local shop in village or nearby town	1.00			1.00		
Wholesale shop	1.98	(1.14, 3.43)		1.65	(0.90, 3.00)	
Local market	1.30	(0.87, 1.94)		0.96	(0.58, 1.57)	
Truckseller/across the border	4.68	(0.96, 22.85)	0.015	3.02	(0.55, 16.73)	0.066
Type of salt package						
Box	1.00					
Plastic bag with label	0.66	(0.37, 1.18)				
Plastic bag without label	1.02	(0.51, 2.06)				
Loose	0.80	(0.43, 1.48)	0.268			
Frequency of buying salt						
Every 1–2 weeks	1.00			1.00		
Once a month	1.34	(0.84, 2.14)		2.89	(1.01, 8.22)	
Once every 2–3 months	1.76	(1.04, 2.98)		2.00	(0.85, 4.75)	
Once every 6 months or less often	1.47	(0.82, 2.64)	0.195	2.26	(1.01, 5.04)	0.040
Quantity of salt bought last time						
< 1 kg	1.00			1.00		
1 kg	2.30	(0.88, 6.03)		2.56	(0.81, 8.14)	
> 1 kg to 5 kg	1.58	(0.72, 3.47)		1.85	(0.72, 4.73)	
≥ 5 kg	2.24	(1.06, 4.72)	0.073	1.96	(0.79, 4.87)	0.128
Storage of salt						
Container with lid	1.00					
Container without lid	1.24	(0.85, 1.79)				
Leave in bag or packet	0.67	(0.22, 2.05)	0.368			
Household head has ever heard about iodized salt	1.26	(0.53, 3.02)	0.589			
Iodization level of salt						
Not iodized	1.00			1.00		
Inadequately iodized (< 15 ppm)	0.56	(0.38, 0.84)		0.72	(0.44, 1.17)	
Adequately iodized (≥ 15 ppm)	0.76	(0.51, 1.14)	0.017	1.25	(0.74, 2.11)	0.055
Thyroglobulin concentration						
Tg 4–40 μg/L	1.00					
Tg < 4 μg/L	1.05	(0.68, 1.62)				
Tg >40 μg/L	1.78	(1.14, 2.81)	0.039			
Urinary iodine concentration (UIC)						
UIC ≥100 μg/L	1.00					
UIC 50–99 μg/L	0.71	(0.44, 1.14)				
UIC <50 μg/L	1.09	(0.70, 1.70)	0.067			

^a^Crude Odds Ratio.

^b^School-level random effect included.

^c^*P*-value based on likelihood ratio test (LRT).

^d^Pathogenic parasites: Helminths (*Hymenolepis nana*, *Ascaris lumbricoides*, Hookworm, *Enterobius vermicularis*, *Trichuris trichiura*, *Fasciola hepatica*, *Hymenolepis diminuta*, *Dicrocoelium dendriticum*), Intestinal protozoon (*Giardia intestinalis*, *Entamoeba histolytica/E. dispar.*

A risk factor remaining significant in the multivariable model was 'buying salt once a month (OR = 2.89, 95% CI = 1.01-8.22) and 'buying salt once every six months or less often’ (OR = 2.26, 95% CI = 1.01-5.04) compared to 'buying salt every 1–2 weeks’.

### Level of urinary iodine concentration

**Figure 2:**
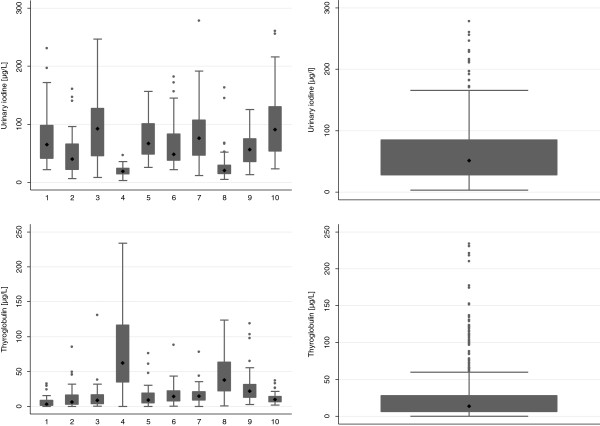
Box plot of median and 25%, 75% percentiles of UIC and serum thyroglobulin in schoolchildren in South Tajikistan, 2009.

Figure 2 displays the median UIC and 25th and 75th percentiles of examined schoolchildren. The overall median UIC was 51.2 μg/L (27.9 μg/L - 85.0 μg/L) indicating 'mild iodine deficiency’ borderline to 'moderate iodine deficiency’. The median UIC in girls (43.3 μg/L, 24.1 μg/L - 75.9 μg/L) was considerably lower than the median UIC in boys (58.4 μg/L, 31.7 μg/L - 92.6 μg/L; Pearson’s χ^2^ = 5.147, *P* = 0.023). Age-related differences were statistically significant, but not showing a trend: the youngest age group of 7–8 years old students had a median UIC of 49.2 μg/L (27.6 μg/L - 82.4 μg/L). Students aged 9 years had the highest median UIC (57.4 μg/L, 30.5 μg/L - 87.5 μg/L) whereas the oldest counterparts of 10–11 years showed the lowest median UIC of 42.0 μg/L (24.2 μg/L - 82.5 μg/L; Pearson’s χ^2^ = 8.594, *P* = 0.014).

The severity of urinary iodine concentration is given in Table 3. Overall, 18.3% of all schoolchildren had adequate iodine nutrition. One third (32.1%; 95% CI = 28.3%-36.0%) had a mild, one third (35.1%; 95% CI = 31.3%-39.2%) a moderate and 14.4% (95% CI = 11.7%-17.5%) had a severe iodine deficiency according to WHO guidelines. Girls were more often moderately to severely iodine deficient than boys (55.1% *versus* 44.4%, respectively, Pearson’s χ^2^ = 6.729, *P* = 0.035).

**Table 3:** Urinary iodine concentrations overall and stratified by sex in South Tajikistan, 2009

**School nr**	**Boys**			**Girls**			**χ**^**2**^^**a**^	***P*-value**	**Total**		
	**< 50 μg/L**	**50-99 μg/L**	**≥100 μg/L**	**< 50 μg/L**	**50-99 μg/L**	**≥100 μg/L**			**< 50 μg/L**	**50-99 μg/L**	**≥100 μg/L**
**1 (61)**	10 (28.6)	13 (37.1)	12 (34.3)	11 (42.3)	12 (46.2)	3 (11.5)	NA^b^	0.131	21 (34.3)	25 (41.0)	15 (24.6)
**2 (59)**	20 (57.1)	12 (34.3)	3 (8.6)	16 (66.7)	8 (33.3)	0	NA^b^	0.407	36 (61.0)	20 (33.9)	3 (5.1)
**3 (59)**	10 (31.3)	8 (25.0)	14 (43.7)	6 (22.2)	10 (37.0)	11 (40.7)	NA^b^	0.537	16 (27.1)	18 (30.5)	25 (42.4)
**4 (63)**	27 (100.0)	0	0	36 (100.0)	0	0	NA^b^	NA	63 (100.0)	0	0
**5 (49)**	6 (31.6)	8 (42.1)	5 (26.3)	9 (30.0)	13 (43.3)	8 (26.7)	NA^b^	0.999	15 (30.6)	21 (42.9)	13 (26.5)
**6 (54)**	15 (50.0)	10 (33.3)	5 (16.7)	13 (54.2)	9 (37.5)	2 (8.3)	NA^b^	0.802	28 (51.9)	19 (35.1)	7 (13.0)
**7 (66)**	5 (15.6)	18 (56.3)	9 (28.1)	15 (44.1)	11 (32.4)	8 (23.5)	NA^b^	0.033	20 (30.3)	29 (43.9)	17 (25.8)
**8 (66)**	28 (87.5)	3 (9.4)	1 (3.1)	31 (91.2)	2 (5.9)	1 (2.9)	NA^b^	0.831	59 (89.4)	5 (7.6)	2 (3.0)
**9 (62)**	9 (23.7)	27 (71.0)	2 (5.3)	15 (62.5)	7 (29.2)	2 (8.3)	NA^b^	0.003	24 (38.7)	34 (54.8)	4 (6.5)
**10 (50)**	5 (20.8)	8 (33.3)	11 (45.8)	5 (19.2)	10 (38.5)	11 (42.3)	NA^b^	0.935	10 (20.0)	18 (36.0)	22 (44.0)
**Total (589)**	135 (44.4)	107 (35.2)	62 (20.4)	157 (55.1)	82 (28.8)	46 (16.1)	6.73	0.035	292 (49.6)	189 (32.1)	108 (18.3)
**95% CI**	(38.7, 50.2)	(29.8, 40.9)	(16.0, 25.4)	(49.1, 61.0)	(23.6, 34.4)	(12.1, 20.9)			(45.5, 53.7)	(28.3, 36.0)	(15.3, 21.7)

^a^Comparison of frequencies using 3 categories '≥100 μg/L’ ’50-99 μg/L’ and '<50 μg/’ using Pearson’s χ^2^ Test; ^b^ = Fisher’s exact test.

### Level of thyroglobulin concentration

The overall median of thyroglobulin of the examined students was slightly elevated at 13.9 μg/L (25th, 75th percentile: 6.2 μg/L and 28.2 μg/L). Median thyroglobulin levels stratified by schools were in the range of 3.0 μg/L (1.3 μg/L, 9.0 μg/L) and 64.6 μg/L (34.7 μg/L, 120.7 μg/L) (Figure 2). A moderate negative correlation was found between thyroglobulin concentration and urinary iodine concentration (Spearman’s rank correlation coefficient ρ = -0.504, *P* ≤ 0.001).

### Salt purchase, storage and knowledge about importance of iodised salt

Salt was obtained from village grocery stores (64.1%), the local market (35.1%) and from nearby wholesale shops in the district centre (19.4%). Few households reported buying salt across the national frontier and from truck sellers. Four out of five households bought salt in labelled and every sixth household in unlabelled plastic bags. Salt was purchased once a month (43.0%) or once every two to three months (26.9%). Almost half of the households bought quantities of more than ten kg, one third between one and five kg and every fourth household bought five to ten kg. Salt was stored in a container with lid or in the original bag.

### Level of salt iodization from households and local selling points

Iodine content of salt samples from households and selling points determined by qualitative and quantitative titration methods are shown in Table 4. The medians of the quantitatively analysed samples ranged between 2.1 ppm and 15.9 ppm for the household salt samples, and between 3.7 ppm and 14.8 ppm for the salt samples from selling points. Overall, one third of the qualitatively determined salt samples from households contained adequate iodine content (32.9%), one third (34.1%) was inadequately and one third not (32.9%) iodized. Iodization levels of salt samples from local selling points showed a similar pattern to the household salt samples: One third (34.9%) were adequately iodized, one third (34.9%) inadequately and one third (30.3%) not iodized. Salt samples from households left in the original packet were less often adequately iodized compared to salt samples from households storing their salt in a container closed with a lid (26.5% *versus* 39.9%, Pearson’s χ^2^ = 9.442, *P* = 0.009).

**Table 4:** Iodine content of salt samples from households and local selling points in South Tajikistan, 2009

**School nr**		**Sample from household (qualitative method)**			**Sample from household (titration method)**		**Sample from local selling point (titration method)**	
		**0 ppm**	**< 15 ppm**	**≥ 15 ppm**				
	**Nr**	**Nr (%)**	**Nr (%)**	**Nr (%)**	**Nr**	**Median (25th, 75th percentile)**	**Nr**	**Median (25th, 75th percentile)**
**1**	61	16 (26.2)	38 (62.3)	7 (11.5)	16	5.3 (2.7, 6.3)	5	14.8 (10.6, 15.9)
**2**	59	25 (42.3)	24 (40.7)	10 (17.0)	15	2.1 (0, 6.3)	4	3.7 (3.2, 4.8)
**3**	59	16 (27.1)	27 (45.8)	16 (27.1)	15	12.7 (5.3, 23.3)	3	10.6 (6.3, 11.6)
**4**	63	57 (90.5)	4 (6.3)	2 (3.2)	16	5.8 (4.2, 10.5)	12	6.1 (5.3, 8.5)
**5**	49	4 (8.2)	17 (34.7)	28 (57.1)	13	6.3 (5.3, 10.6)	8	6.4 (5.3, 10.1)
**6**	54	19 (35.2)	25 (46.3)	10 (18.5)	14	5.8 (0, 10.6)	9	6.3 (6.3, 10.6)
**7**	66	4 (6.1)	20 (30.3)	42 (63.6)	17	15.9 (10.6, 37)	7	10.6 (6.3, 14.8)
**8**	66	38 (57.6)	12 (18.2)	16 (24.2)	17	5.3 (4.2, 10.6)	11	7.4 (5.3, 10.6)
**9**	62	10 (16.1)	27 (43.6)	25 (40.3)	14	5.3 (5.3, 6.3)	2	8.0 (5.3, 10.6)
**10**	50	5 (10.0)	7 (14.0)	38 (76.0)	12	3.7 (0, 12.7)	4	4.8 (4.2, 8.0)
**Total**	589	194 (32.9)	201 (34.1)	194 (32.9)	149	5.3 (4.1, 11.6)	65	7.4 (5.3, 10.6)

## Discussion

Published evidence on prevalence of IDD in children in Tajikistan and other countries of Central Asia over the past decade is scarce and concentrated to the early 2000s. Available epidemiological studies (Table 5) point to a high prevalence of IDD in different regions of Kyrgyzstan, Tajikistan and Uzbekistan in 2002 and 2003.

**Table 5:** Comparison of iodine deficiency indicators between 2000 and 2013 in countries of Central Asia

**Indicator**		**Age range in years**	**sample size n**	**Country**	**Year of survey**	**Source**
**Goitre (overall prevalence %)**	70	all ages	NA (population-based survey)	Surkhandarya region, Uzbekistan	2002^a^	[34]
	65	all ages	NA (population-based survey)	Kashkadarya region, Uzbekistan	2002^a^	[34]
	20-28	9-10	4184	Kyrgyzstan (national)	2003^a^	[35]
	70	9-10	NA	Djalal Abad region, Kyrgyzstan	2003^a^	[35]
	12	8-11	400	Kabul, Afghanistan	2005	[21]
	47	7-11	589	Khatlon region, Tajikistan	2009	Present study
**Urinary iodine concentration (median [μg/L])**	25	9-10	NA	Djalal-Abad region, Kyrgyzstan	2003^a^	[35]
	105	2-15	40 households	Kazakhstan	2003	[7]
	29	2-15	40 households	Tajikistan	2003	[7]
	109	2-15	40 households	Uzbekistan	2003	[7]
	65	0.5-5	2000	Khatlon region, Tajikistan	2003	[36]
	67	8-11	400	Kabul, Afghanistan	2005	[21]
	114	8-10	580	Kyrgyzstan	2007	[37]
	170	NA	879	Turkmenistan	2006^a^	[38]
	72	0.5-5	422	Region of Khatlon, Tajikistan	2009	[39]
	51	7-11	589	Khatlon region, Tajikistan	2009	Present study
**Iodine content of household salt (median [ppm])**	10.5	NA	580	Kyrgyzstan	2007	[37]
	5.3	NA	149	Region of Khatlon, Tajikistan	2009	Present study

^a^Date of publication because the exact date of survey could not be extracted.

Our data demonstrate no distinct changes in the prevalence of goitre in the regions of Khatlon and RRS in the South during the past years, indicating that IDD remain a serious public health issue in this region. In our study, almost every second schoolchild was affected by goitre and every sixth schoolchild suffered from goitre grade 2. The UIC was sufficient in less than 20% of the examined schoolchildren and the median UIC was <100 μg/L in all age classes and schools. Median thyroglobulin concentration was modestly elevated in the overall sample, correlated negatively with UIC and was strongly elevated in schools with the most severe iodine deficiency. One third of the salt samples were not iodised, one third insufficiently and one third inadequately. These findings strengthen the results from the national micronutrient status survey in Tajikistan conducted in 2009 among women in childbearing age and in children aged 6–59 months indicating no improvement of ID indicators between 2003 and 2009 [36,39].

Identified risk factors and protective factors for goitre should be interpreted with caution because of the cross-sectional design of the study. However, three key findings are underlined in the paragraphs below.

Children from households buying less frequently salt were more likely at risk of goitre compared to children from households buying frequently salt (i.e. every one or two weeks). This finding indicates that households buying less frequently salt buy larger quantities. In the bivariate models buying large quantities of salt ≥ 5 kg was a risk factor. Large quantities of salt are assumed to be rather likely unpacked and unlabelled and consequently unprocessed. Moreover, the iodine content in iodized salt vanishes over time, which might be, coupled with storage conditions, of relevance for large quantities bought. Storing the salt in a container with lid was significantly associated with higher iodine content. Storage of salt in open containers was reported as a risk factor in a study from Iran [40].

In our study, drinking water from a public tap emerged as a protective factor compared to surface water used as drinking water source. Other studies assessed potential associations between drinking water and iodine status. A study from Ethiopia indicated that drinking water contaminated with *E. coli* and coliforms were associated with a higher incidence of endemic goitre [41]. High nitrate levels in drinking water were reported to play a role in an increased risk of thyroid dysfunction in a study from Bulgaria [42]. No significant positive association between tap water iodine concentration and UIC among schoolgirls was reported from UK [43].

Children from the socioeconomic middle class were less likely at risk of developing goitre when compared to children from the lowest socioeconomic class, a finding which is supported from previous research. A study on iodine deficiency in schoolchildren in Istanbul revealed a higher prevalence of iodine deficiency in students with less educated caretakers and from poorer families [44]. A cohort study from Denmark observed that lowest education levels were associated with high thyroid volume [45]. Association between high educational level and lower risk of low iodine intake in adults were found in a study from France [46].

One third of the cooking salt samples from households and selling points were not iodised, one third insufficiently, and one third were adequately iodised. Findings from the national micronutrient survey showed for Khatlon that iodization was adequate in more than half of the household salt samples but every fourth salt sample had no iodine [39].

Highest rates non-iodized salt from households and selling points were found in those schools located furthest away from the district centre. In contrast, highest rates of adequately iodized cooking salt and lowest frequencies of goitre were observed in those schools in schools closest to the capital. Remoteness of villages can play a role in accessing iodised salt and exposure to sensitization on prevention of IDD [47].

According to local authorities, the high rate of non-iodised household cooking salt is mainly because of supply from near salt mines and unprocessed salt sold by individuals and small enterprises. The iodine content may also considerably vary in salt samples originating from the same brand and factory due to poor control standards. A study from Kyrgyzstan showed that unpacked salt is up to one third cheaper than packed salt [48]. Persisting insufficiently iodised salt at retailer and household level is partially due to producing of salt with no iodine or varying iodine levels, falsified copies of labelled packets of big producers, and unregulated natural deposits and illegal imports [37,49].

In absence of a functioning monitoring system of universal salt iodisation regulations, empowering of communities and retailers for monitoring iodine content of salt proved to be successful in Kyrgyzstan [6]. The Swiss Health Reform and Family Medicine Support Project (Project Sino) together with community groups of pilot districts adopted key activities to raise awareness on medical and cultural consequences of iodine deficiency. Collaboration between medical staff from primary healthcare centres and district endocrinology services were reinforced and communities supplied with iodine rapid test kits. However, progress of interventions towards universal salt iodation is probably visible only at medium term because of persisting delayed effects of iodine deficiency.

The study suffered from a few methodological and other limitations. Goitre palpation method was used because electricity supply was unreliable and skilled staff and local equipment was not available to perform thyroid ultrasonography. Limitations of palpation methods are intra- and inter-observer variation and risk of misclassification [50]. One experienced endocrinologist was screening the children minimising the risk of inter-observer variation.

There is substantial day-to-day variation of individual iodine intakes and urinary iodine concentrations [1]. Only a single urinary sample was collected and examined in our study owing to logistical constraints. The results of the urinary iodine levels should thus be interpreted with caution. A limitation of the sampling approach used based on a WHO rapid appraisal methodology is the underrepresentation of small schools. Referring to the eligibility of study participants, estimations on the prevalence of goitre might be underestimated because only children attending school were included in the study. It was reported from other studies in Ethiopia that goitrous children are not sent to school by their caretakers because of stigma [51].

## Conclusion

Our study showed that prevalence of IDD represented a serious health issue among schoolchildren in South Tajikistan in 2009. Despite control interventions by the government of Tajikistan and international partners, high rates of goitre are persisting, fitting in the broader picture of identified studies in Tajikistan and neighbouring countries. Households buying frequently salt were less likely at risk of long term effects of ID, and proper storage of salt was significantly associated with higher iodine content. Considering long-term effects such as impaired intellectual function in children [1], and salt iodization as an effective low-cost intervention, concerted efforts to increase consumer and household demand for iodised salt via awareness raising are recommended. Quality of iodine content needs to be monitored at all levels of the system.

## Abbreviations

GDP: Gross domestic product; ICCIDD: International Council for the Control of Iodine Deficiency Disorders; ID: Iodine deficiency; IDD: Iodine deficiency disorders; JPM: Joint Monitoring Programme for Water Supply and Sanitation by the WHO and UNICEF; LRT: Likelihood ratio test; MoH: Ministry of Health; ppm: Parts per million; Project Sino: Swiss Health Reform and Family Medicine Support Project; RRS: Region of Republican Subordination; SD: Standard deviation; SSESS: State Sanitary Epidemiological Surveillance Service; UIC: Urinary iodine concentration; WHO: World Health Organization.

## Competing interests

The authors declare that they have no competing interests.

## Authors’ contributions

BM and KW conceived and MZ contributed to further development of the study design. MD, GK and VJ provided inputs to the methodology. Field data collection was coordinated by BM with assistance of MD, GK and VJ. BM performed data analysis and drafted the manuscript which was revised by GK, MZ and KW. All authors read and approved the final manuscript.

## Pre-publication history

The pre-publication history for this paper can be accessed here:

http://www.biomedcentral.com/1472-6823/13/50/prepub
